# Opportunistic infections in HIV positive patients in Bahrain in 4 years study 2009-2013

**DOI:** 10.1186/1471-2334-14-S2-P54

**Published:** 2014-05-23

**Authors:** Nermin Saeed, Eman Farid, Afaf E Jamsheer

**Affiliations:** 1 SMC, Pathology Department, Microbiology Section, Muharaq, Bahrain

## Objectives

This study aimed to examine the prevalence of opportunistic infections in HIV-infected patients in Bahrain and related them to the absolute CD4 count, CD4% and CD4/CD8 ratio.

## Methods

The research was a retrospective, cohort study using laboratory records from a major hospital in Bahrain, from January 2009 to May 2013. Opportunistic infections (OIs) and absolute CD4 count, CD4% and CD4/CD8 ratio were recorded for every patient.

## Results

CD4% and absolute CD4 count in HIV patients with associated infections was significantly lower than in those without associated infections (P< 0.001) but there was no significant difference in CD4/CD8 Ratio between the two groups. Infection with Staphylococcus aureus was the commonest encountered infections and present in 9.8% % of total AIDS patients and 28.7% of AIDS patient group who had OIs; followed by yeast infections (9.2% and 27.2% respectively). Mycobacterium tuberculosis was present in 3.6% of total AIDS patients and 10.6% of the group with OIs while Mycobacterium Other Than Tuberculosis (MOTT) was present in 2.5% and 7.5% respectively. Pneumocystis jirovecii pneumonia (PCP) was observed in 5.1% and 15.1% respectively. The least bacterial infections observed were Streptococcus penumoniae, Streptococcus melleri, Stenotrophomonas maltophilia, & Citrobacter species. Herpes simplex II (HSV-II) was the commonest OIs observed while Cytomegalovirus antigenemia was only present in 2% and 6% respectively.

## Conclusion

Studying the pattern of OIs in HIV-infected patients in Bahrain is of paramount importance due to scarcity of data in the Arab worlds. This help to improve physician's awareness to improve care of AIDS patients.

